# Burnout, Compassion Fatigue, and the Long Haul of Caring for Long COVID

**DOI:** 10.1093/ofid/ofae080

**Published:** 2024-02-07

**Authors:** Christian B Ramers, John D Scott, Bruce B Struminger

**Affiliations:** Family Health Centers of San Diego, Laura Rodriguez Research Institute, San Diego, California, USA; School of Medicine, UC San Diego, San Diego, California, USA; School of Public Health, San Diego State University, San Diego, California, USA; Global Hepatitis & COVID-19 Treatment Access Program, Clinton Health Access Initiative, Boston, Massachusetts, USA; Department of Medicine, University of Washington, Seattle, Washington, USA; Rehabilitation Clinic, Harborview Medical Center, Seattle, Washington, USA; Project ECHO, University of New Mexico Health Sciences Center, Albuquerque, New Mexico, USA

**Keywords:** long COVID, myalgic encephalomyelitis/chronic fatigue syndrome (ME/CFS), post COVID-19 conditions (PCCs)

## Abstract

The current landscape of clinician burnout is prompting the need for our health care system to revise its approach toward complex conditions such as long coronavirus disease (COVID), myalgic encephalomyelitis/chronic fatigue syndrome (ME/CFS), and other postinfectious fatiguing illnesses (PIFIs). We discuss our efforts here at Family Health Center of San Diego (FHCSD) to help share insight and glean perspective from clinicians who have participated in our Centers for Disease Control and Prevention (CDC)–funded 3-year continuing professional development initiative. The Long COVID and Fatiguing Illness Recovery Program uses multidisciplinary team-based case consultation and peer-to-peer sharing of emerging best and promising practices (ie, teleECHO [Extension for Community Healthcare Outcomes]) to support the management of complex cases associated with long COVID, ME/CFS, and other PIFIs. We believe that this perspective captures a key moment in the trajectory of postpandemic clinician burnout and prompts further reflection and action from the health care system to improve clinician- and patient-level outcomes related to the care of patients with postinfectious fatiguing illnesses.

As the US health care system and its weary foot soldiers emerge from the 3-year pandemic state of emergency, a daunting challenge remains: caring for the millions of Americans afflicted by post-COVID conditions (PCCs), commonly known as long coronavirus disease (COVID). This is occurring at a time when symptoms of burnout among physicians persist, and health care systems continue to be strained by high patient volumes and staffing challenges [1]. In the wake of pandemic stress, high workloads, and ongoing frustrations, many health care workers are choosing to retire early or leave their professions altogether. According to recent reports, 52% of nurses and 20% of doctors are planning to leave their clinical practice [2].

Post-COVID conditions are a heterogeneous group of physical and mental symptoms that persist for at least 4 weeks following a severe acute respiratory syndrome coronavirus 2 (SARS-CoV-2) infection. Patients often suffer 1 or more overlapping syndromes, including cognitive disturbances, fatigue and postexertional malaise, autonomic dysfunction with orthostatic intolerance, or other skin, gastrointestinal, or neurologic manifestations. Despite sometimes disabling symptoms, routine diagnostic testing often yields normal results, which can prompt uncomfortable interactions between patients and clinicians who are faced with the subjective complaints, but often without supportive evidence of objective pathology. Unfortunately, many long COVID patients are told their symptoms “aren’t real” or, just as damaging, “are all in their heads” [3]. Sadly, these reports of patients having their concerns dismissed hint at a weakness in our current system to properly manage symptoms that do not fit into traditional diagnostic paradigms.

Long COVID may be the most recent postinfectious syndrome, but it is not the first and is unlikely to be the last. Fatiguing illnesses have been described in association with numerous other infectious diseases—Epstein Barr virus, varicella virus, *Borrelia burgdorferi* (Lyme disease), and West Nile virus [4]. Additionally, myalgic encephalomyelitis/chronic fatigue syndrome (ME/CFS), a serious and debilitating condition, shares many features with more severe long COVID and may share underlying pathophysiology [5]. While we await the findings of the National Institutes of Health's RECOVER COVID Initiative [6], we must expand the diagnostic lexicon to include all postinfectious fatiguing conditions. Unfortunately, as with long COVID, many of these conditions are dismissed as lacking legitimacy due to lack of a clear case definition, understanding of the pathophysiology, and effective treatment options, creating tension between a weary medical workforce and an often “gaslit” and suffering patient population.

Our current system of siloed, efficiency-oriented medical care is ill-equipped to adequately address such complex chronic conditions. Furthermore, COVID-related health disparities are exacerbated by poor health care access, impacted primary care schedules, and long waitlists for the few academic medical center–based multidisciplinary long COVID clinics that exist. On the front lines of long COVID in primary care clinics around the country, well-meaning but overworked clinicians, often frustrated that they can offer no or inadequate meaningful help in a 15-minute visit, struggle to attend to the needs of desperate patients. To effectively address this ongoing crisis, we must rethink how we approach emerging, often poorly understood complex chronic diseases, particularly in the domain of postinfectious fatiguing illnesses. Several alternative models are being tried, each with their own merits and challenges [7, 8].

In December 2021, supported by the CDC, we launched the Long COVID & Fatiguing Illnesses Recovery Program (LC&FIRP) to bring multidisciplinary specialty expertise to an underserved, primary care setting. Using the ECHO case-based tele-mentoring model, this continuing professional development program has the dual aims of improving patient-reported outcomes of physical, emotional, and mental health in patients with long COVID and other postinfectious fatiguing illnesses and improving the satisfaction, self-efficacy, and engagement of frontline providers within a Federally Qualified Health Center (FQHC) network. Weekly tele-mentoring sessions include a multidisciplinary panel of clinical experts from across the United States, a small cohort of local primary care providers, and always a person with lived experience to elevate the knowledge and insights of the patient experience. Separately, monthly webinars are conducted that are open to a global audience in which the latest scientific literature, lessons learned, and emerging best and promising practices are discussed [9]. To date, 3651 individuals from nearly all 50 states and 59 countries have attended the monthly learning sessions. A CDC-commissioned qualitative study of providers in the weekly tele-mentoring sessions revealed that providers reported increased awareness of long COVID, more confidence in their ability to care for their patients, more empathy, increased patience, and the ability to provide more treatment options. A detailed quantitative and qualitative analysis of patient and provider experiences in the program is forthcoming, as well as the results of a randomized controlled trial comparing the patient outcomes across cohorts of providers who either participate in the weekly case-based telementoring sessions or do not have that opportunity, as well as a comparison with outcomes of patients who attend an academic medical center long COVID multispecialty clinic. Despite the frustrations of caring for patients with complex medical and sociological challenges, hope for better understanding and more evidence-based and patient-responsive care is emerging. Providing community-based primary care providers with routine access to multidisciplinary experts along with access to their peers for collaborative problem-solving, thereby supporting their ability to better care for struggling patients, is a powerful antidote to the fatigue and burnout that has affected so many providers.

Continued basic research remains critical to inform effective treatments for long COVID and other postinfectious fatiguing illnesses, and alternate care models beyond the 15-minute visit should be explored. However, a more immediate goal must be improving education and awareness and collaborative problem-solving among providers and encouraging symptomatic treatment when symptoms persist that are otherwise medically unexplained [10]. Meeting the emotional needs of patients who have lost their identity, experienced trauma at the hands of medical professionals, and struggle to cope with a condition not widely recognized by medical providers starts with the simple phrase, “I hear you and believe you.” After acknowledging the patient's suffering, clinicians are often left not knowing what to do for them; therefore, educational resources, including routine access to multidisciplinary expertise and peer-to-peer collaborative problem-solving, and the time to take advantage of them are important. And while there are currently no approved treatments for long COVID, management strategies developed for ME/CFS patients can be used for long COVID patients with similar symptoms.

Efforts to understand the unfolding long COVID crisis may offer fresh insights and support for clinicians caring for patients who suffer from fatiguing illnesses associated with other infections. Beyond acknowledging that fatiguing conditions exist, we must commit to their care, improve coordination among frontline providers and specialists, and accelerate clinical trials of new or repurposed therapeutics. A rethinking of the 15-minute visit with more supportive case management is needed. Ultimately, it will take collaboration between public health entities, medical schools, medical societies, payers, researchers, patients, and advocates to optimize care for this poorly understood yet highly prevalent condition. And to stave off both the scourge of provider burnout and the pressing volume of long COVID patients, we must increase our collective awareness, tap into and cultivate our deepest wells of empathy, and advocate for systemic and policy change.
